# A Tailored Preparation Method of Variable Strength for Ultra-High-Strength Steel Sheet and Mapping Mechanism between Process and Property

**DOI:** 10.3390/ma15196620

**Published:** 2022-09-23

**Authors:** Guo-Zheng Quan, Yan-Ze Yu, Yu Zhang, Yu-Qing Zhang, Wei Xiong

**Affiliations:** 1 Chongqing Key Laboratory of Advanced Mold Intelligent Manufacturing, School of Material Science and Engineering, Chongqing University, Chongqing 400044, China; 2 State Key Laboratory of Materials Processing and Die & Mould Technology, Huazhong University of Science and Technology, Wuhan 430074, China; 3 Key Laboratory of Advanced Reactor Engineering and Safety of Ministry of Education, Collaborative Innovation Center of Advanced Nuclear Energy Technology, Institute of Nuclear and New Energy Technology, Tsinghua University, Beijing 100084, China

**Keywords:** phase transformation, ultra-high-strength steel, preparation method, mapping mechanism

## Abstract

The spatiotemporal phase transformation during hot stamping would considerably effect the microstructure and mechanical properties of steels. In order to manufacture hot-stamping components of ultra-high-strength steel with tailored mechanical properties, the effect of the quenching time and the temperature of the die on the phase-transformation characteristics and mechanical property of ultra-high-strength steel was deeply studied. A finite element (FE) model coupled with a thermomechanical phase was employed to perform a succession of simulations for hot stamping corresponding to different quenching times and the temperatures of die, and the corresponding hot stamping experiments were performed. The 3D mapping surfaces of the temperature; quenching time; and three microstructures, namely austenite, bainite, and martensite, were constructed, and the mapping relationships in such surfaces were further explained by microstructural observations. Subsequently, based on the test results of the mechanical properties, the relationship curves of hardness and tensile strength, hardness, and elongation at break were fitted respectively, and then the 3D mapping surfaces were constructed for hardness, tensile strength, and elongation at break, which varied with the temperature die and quenching time. Finally, the quenching parameters of the automobile B-pillar were designed according to the constructed mapping relationship, and the hot-stamping FE simulation of the automobile B-pillar was developed. The result shows that those constructed mapping surfaces are helpful for adjusting the local mechanical property of the steels by designing the parameters.

## 1. Introduction

**Figure 1:**
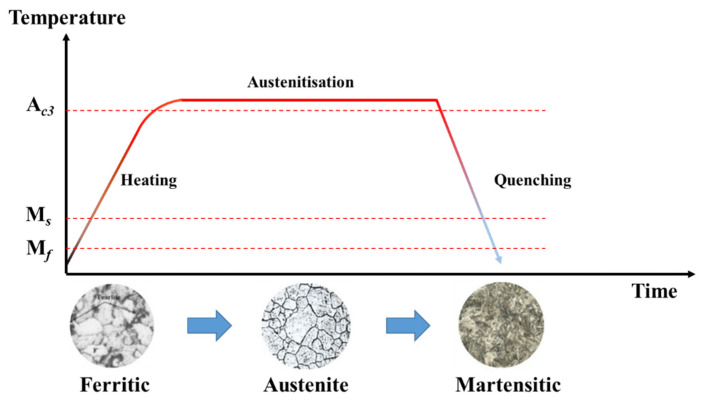
Hot-stamping process of UHSS.

**Figure 2:**
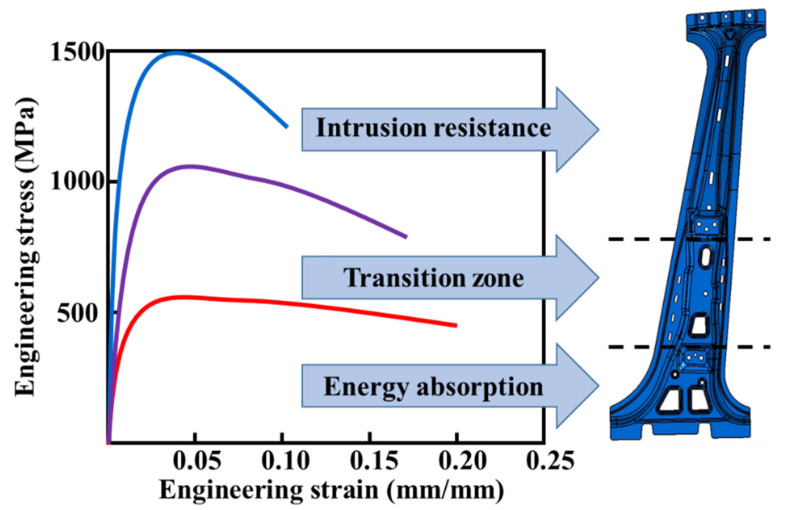
Schematic of the B-pillar with different properties.

With the increasing requirements for energy-saving and safety, ultra-high-strength steel (UHSS) with excellent comprehensive properties of strength, toughness, ductility, and fatigue is widely used in the automobile industry [1,2,3]. Hot stamping is the most effective method to produce UHSS components [4,5]. The conventional hot-stamping process of UHSS is shown in Figure 1. Firstly, the UHSS sheet was heated up to the austenitizing temperature (A*_C3_*) and held for a certain time, so that the original ferrite was completely transformed into uniform austenite. Then the sheet was transferred to the die for hot forming. Meanwhile, the temperature of die was dropped through the water-cooling system to obtain a large temperature gradient between the die and sheet, and a holding pressure was continually applied on the sheet to ensure sufficient contact between the die and sheet [6]. Finally, the martensite transformation of the sheet was completed during the quenching process, and components with a fully martensitic structure were obtained. Although 100% martensite provides extreme strength for components, some components (such as the automobile B-pillar) also simultaneously demand the zone with high ductility to meet the impact energy absorption requirements [7,8,9], as shown in Figure 2. That is, it is difficult for the components with a single mechanical property to simultaneously satisfy the requirements of different serving conditions, as this greatly limits the application of the hot stamping of UHSS. Therefore, it is urgent to design the mechanical properties of components according to their serving conditions.

Nowadays, many researchers have devoted their time to modifying the hot-stamping process to manufacture the component with different mechanical properties. The differential heating and cooling strategies, the tool with varied thermal conductivity, structured tool surface, and integrated partial contact gaps were used to study the variation of mechanical properties of UHSS during hot stamping by Maikranz Valentin et al. [7], Casas et al. [10], Mori et al. [11], and George et al. [12]. Although these studies indicate that the local adjustment of mechanical properties can be achieved by changing the phase transformation during the quenching process, it is an intricate issue of obtaining the desired microstructure and then obtaining the desired mechanical properties through the accurate design of the quenching parameter. Consequently, a detailed description of the correlation mechanism between the quenching parameters and phase-transformation characteristics during hot-stamping quenching is proposed. However, it is a valuable and difficult issue.

In recent years, numerous researchers have focused on the description of phase-transformation characteristics during hot-stamping quenching. Bardelcik et al. [13] found that the microstructures evolved from bainite to martensite when usibors 1500 p were quenched at a cooling rate of 14 K/s to 50 K/s. Neumann et al. [14] simulated the hot-stamping process of 22MnB5 steel under different cooling conditions and determined that the quenching time had the greatest impact on the final microstructure and mechanical properties of the component. Li et al. [15] simulated the hot-stamping process of boron steels by ABAQUS/Explicit and indicated that the lower deformation temperature and higher strain rate were more conducive to diffusion transformation. Zhou et al. [16] analyzed the microstructure evolution of steel 22MnB5 during the hot-stamping process with partial heating by finite element (FE) method, and the result showed that almost complete martensite was obtained in the high-temperature zone, and the mixed state of ferrite and pearlite was obtained in the low-temperature zone. In conclusion, these investigations illustrated the effect of different processing parameters on the phase transformation, among which quenching time and the temperature of die have a greater effect. Nevertheless, these works were based on one processing parameter, without considering the effect of parameter change history and multi-parameter interaction on the phase transition, which is essential for understanding and even designing the dynamic and spatiotemporal phase transformation in the hot-stamping process. To introduce the effect caused by parameter-change history and multi-parameter interaction during hot stamping into the description work, numerous efforts have been made by researchers. Wang et al. [17] compared the distribution characteristics of the final retained austenite under different heating histories through FEM, and the result of simulations was verified by experiments. Tang et al. [18] characterized the microstructure of hot-stamped B1500HS under different deformation histories, and the result indicated that the higher the real strain, the lower martensite transformation temperature. However, these studies lack the description of the corresponding relationship between specific quenching parameters and a specific microstructure, and it is hard to precisely determine the quenching parameters corresponding to the desires. It means that the previous method is unable to design the quenching parameter corresponding to the final desired microstructure precisely. Furthermore, the abovementioned approach does not support the quenching parameters’ design according to mechanical properties, i.e., the strength, hardness, and elongation, a class of mechanical property indexes determined by microstructures.

The hot-stamping quenching parameters determine the microstructures and distributions of UHSS, which, in turn, determine the mechanical properties. Therefore, in order to obtain the components of UHSS with different mechanical properties through quenching parameter design, it is a prerequisite to clarify the relationship between phase transformation and multiple parameters during hot-stamping quenching. In the present study, the FEM coupling thermomechanical phase was employed to reveal the effect of the temperature of the die and quenching time on the phase-transformation characteristic of UHSS during hot-stamping quenching. Meanwhile, the hot-stamping-sheet experiment of UHSS was carried out, and the effect mechanism was further explained by microstructure characterization and mechanical property tests. After that, the mapping relationship of quenching parameters, phases, and mechanical properties was constructed. These mapping relationships can be effectively applied in the quenching-parameter design of components with variable strength to satisfy the requirements of different serving conditions. Finally, based on the mapping relationship, the quenching parameters of the automobile B-pillar were designed, and the FE simulation was carried out.

## 2. Experimental Materials and Methods

**Figure 3:**
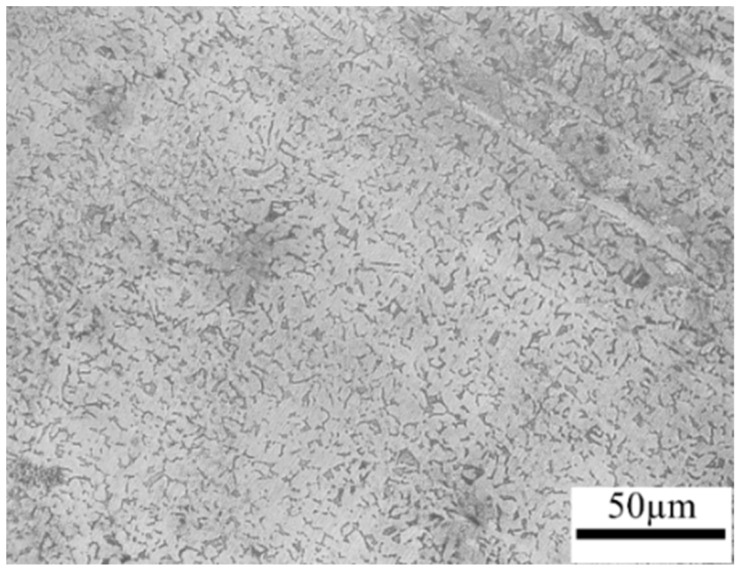
Initial microstructure of BR1500HS.

**Figure 4:**
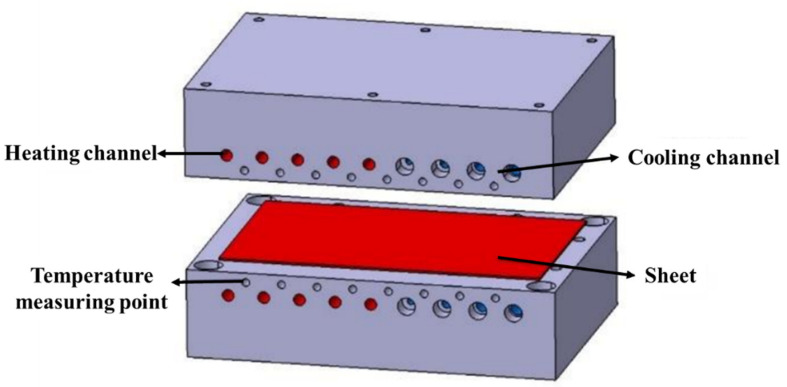
Schematic of the temperature-control die.

The studied steel in this work was boron steel BR1500HS, whose chemical composition was given in Table 1. The steel specimens were received from a hot-rolled sheet manufactured by the China Baowu Steel Group Corporation Limited (Shanghai, China); the initial material of BR1500HS shows a pearlitic and ferritic microstructure, as shown in Figure 3. The hot-stamping experiments of UHSS steel were performed in the hydraulic-forming press. The hot-stamping experimental apparatus mainly comprises a hydraulic forming press, a temperature-control die, and a temperature-control system. Figure 4 shows the schematic of the temperature-control die. The geometry of the sheet was rectangular, with a length of 270 mm, a width of 160 mm, and a thickness of 1.8 mm. The heating channels were arranged on the left side of the temperature-control die, and the cooling channels were arranged on the right side of the temperature-control die. The temperature-detection-and-control system was equipped to dynamically adjust the temperature change in the die surface. At the same time, eight temperature measuring points were chosen to monitor and control the temperature of the die. Firstly, the sheet was austenitized at 950 °C in the resistance heating furnace for 5 min and transferred into the die for hot forming. Then the temperature of the die was adjusted to the designed temperature, using the temperature-control system. Finally, the sheet was quenched and cooled after forming. The quenching parameters of the hot stamping-experiment are listed in Table 2.

**Figure 5:**
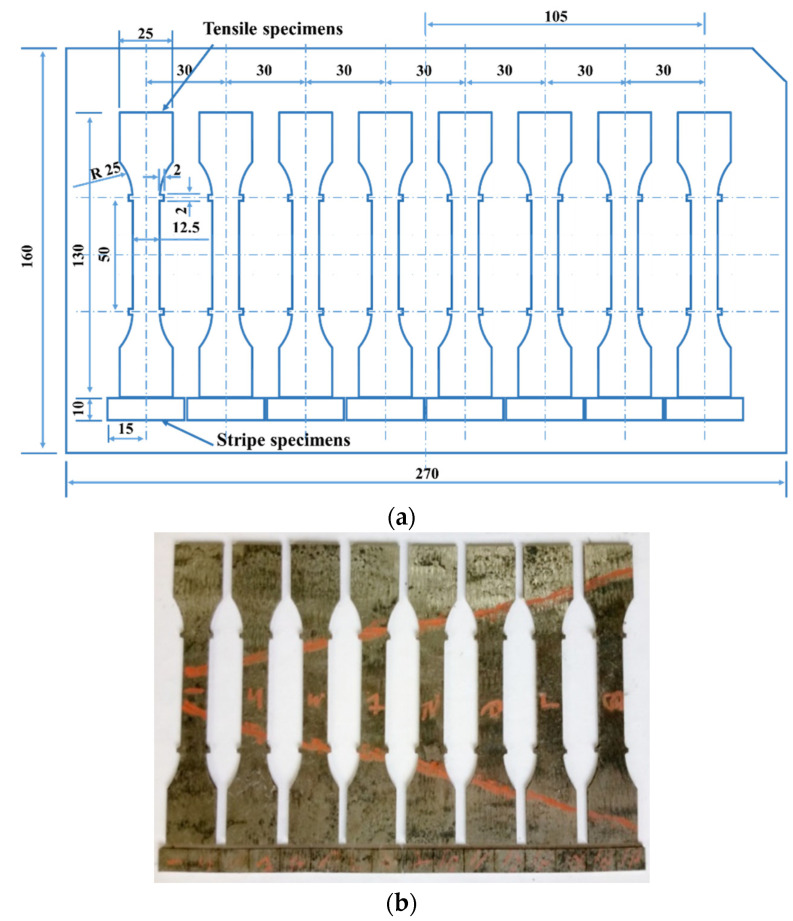
(**a**) Location and (**b**) entity of specimens.

**Figure 6:**
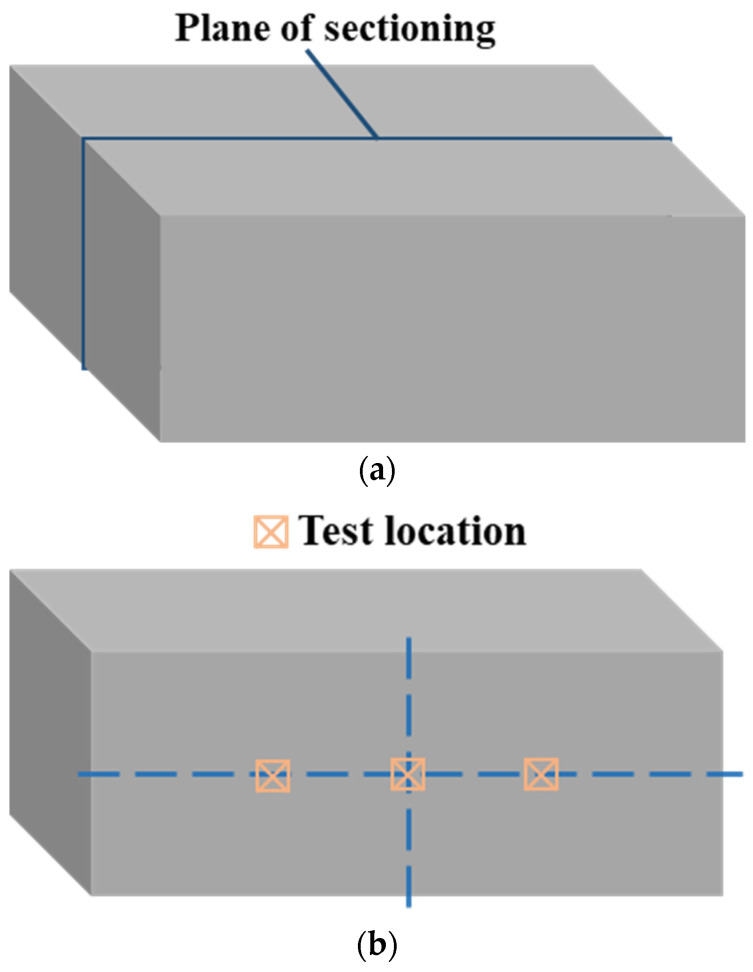

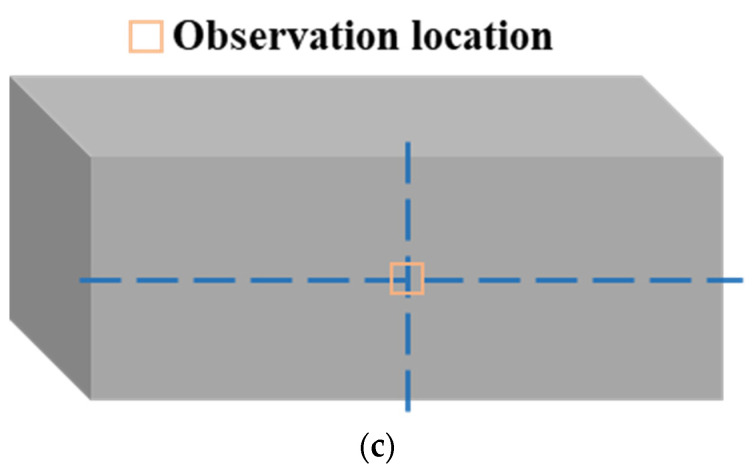
Schematic representation for (**a**) cutting plane of stripe specimen, (**b**) locations for microhardness test, and (**c**) locations for microstructure observation.

In order to test the tensile strength, hardness, and elongation at break of the sheet at each temperature measuring point, a series of tensile tests and microhardness tests were performed. After the hot-stamping experiment, along the temperature gradient direction, a series of tensile specimens were cut by Wire Electrical Discharge Machining (WEDM) for tensile test. Meanwhile, the stripe specimen close to the tensile specimen was also cut for the microhardness test, as shown in Figure 5. The electromechanical universal testing machine was employed to carry out the tensile test of tensile specimens at room temperature and a strain rate of 0.001 s^−1^, and then the stress–strain curve of the specimen was obtained. The microhardness tester was used to conduct the microhardness test. The stripe specimen was sectioned along the center perpendicular to the length direction, and the one-side cut-surfaces of stripe specimens were grinded with abrasive papers and polished, as shown in Figure 6. The hardness of the three points was measured along the center line of the stripe specimen under a loading force of 1000 g and loading time of 10 s, as shown in Figure 6, and the average value was taken as the final hardness index.

In order to characterize the microstructure of the sheet at each temperature measuring point, the other side cut-surfaces of stripe specimens were grinded with grinding paper and polished. In the following, the cut-surface was etched with 4% nitric acid alcohol solution. Finally, the microstructures of the stripe specimens were observed by the optical microscopy.

## 3. Clarification of the Mapping Relationship in Conjunction with Microstructure Characterization and Tensile Test

### 3.1. Effect of Quenching Time and the Temperature of Die on Phase Transformation

**Figure 7:**
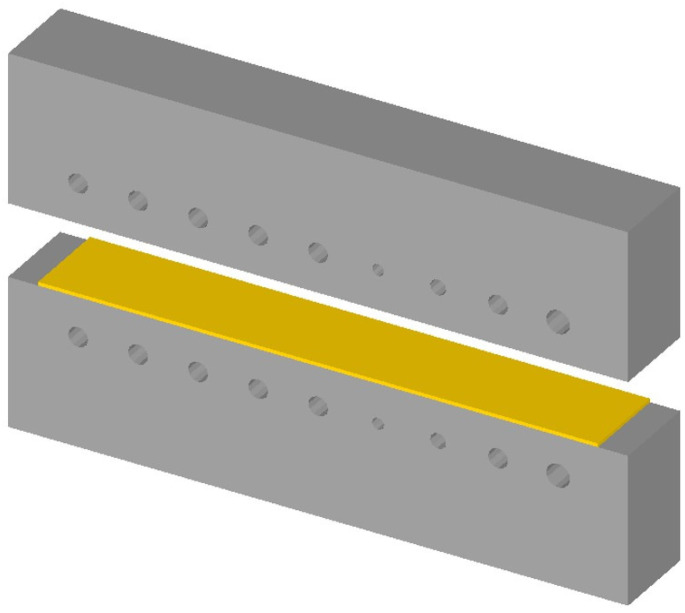
Finite-element model of hot stamping.

**Figure 8:**
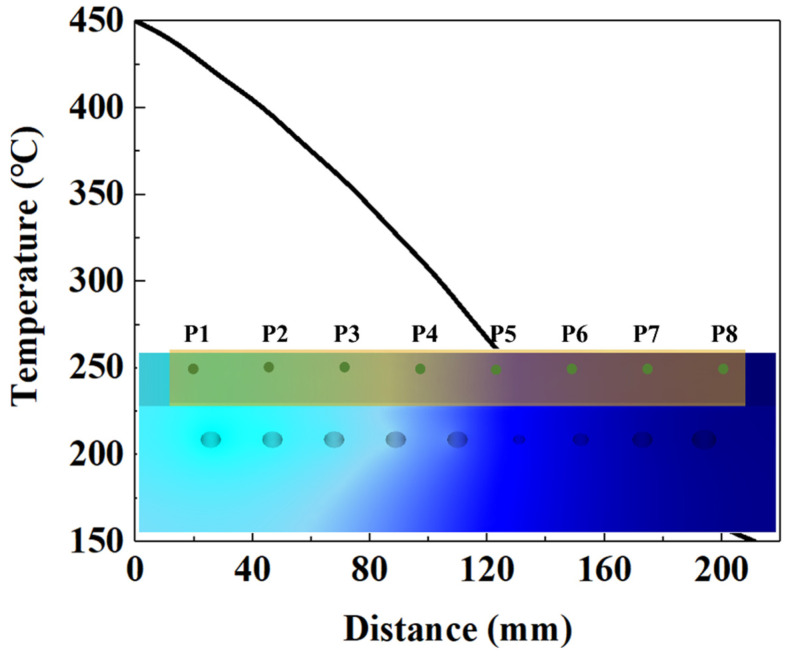
Temperature distribution of die surface and eight temperature measuring points of sheet.

In order to reveal the effect of the quenching time and temperature of the die on the phase-transformation process of UHSS, a thermal-force-phase-coupling model of the hot-stamping process was constructed in DEFORM-3D 11.0 simulation software. Since the die structure was symmetrical, in the FE model, the die structure was simplified, and 1/4 of it was taken for modeling, as shown in Figure 7. Based on the quadrilateral mesh method, the top die and bottom die were meshed as 100,000 elements, respectively, and the sheet was meshed as 25,000 elements. The heating temperature of the sheet was 950 °C. The friction coefficient was 0.3, the holding pressure was 30,000 N, and the pressing speed of the top die was 15 mm/s. In our previous research [19], the multi-phase transformation kinetics model of BR1500HS was developed as follows:

$$V=1-\exp\left[-0.02489*\left(420-T\right)\right]$$

where *V* represents the volume fraction of martensite. Here, this model was embedded into DEFORM-3D software to uncover the phase-transformation process. The relevant parameters of BR1500HS, namely the Young’s modulus, Poisson ratio, and thermal conductivity, came from the material parameters in the Deform material library, as listed in Table 3. The simulation quenching parameters were set according to the hot-stamping scheme in Table 2. In this FE simulation, heating and cooling were realized by applying equivalent heat flow and different heat-transfer coefficients on the pipe’s surface, respectively. After heating, the temperature distribution of the die surface was close to the preset temperature distribution, as shown in Figure 8. For the sake of revealing the phase-transformation characteristic of the sheet during hot stamping, eight points of the sheet were chosen from Figure 8, and the temperature and phase-transformation process of eight points were monitored.

**Figure 9:**
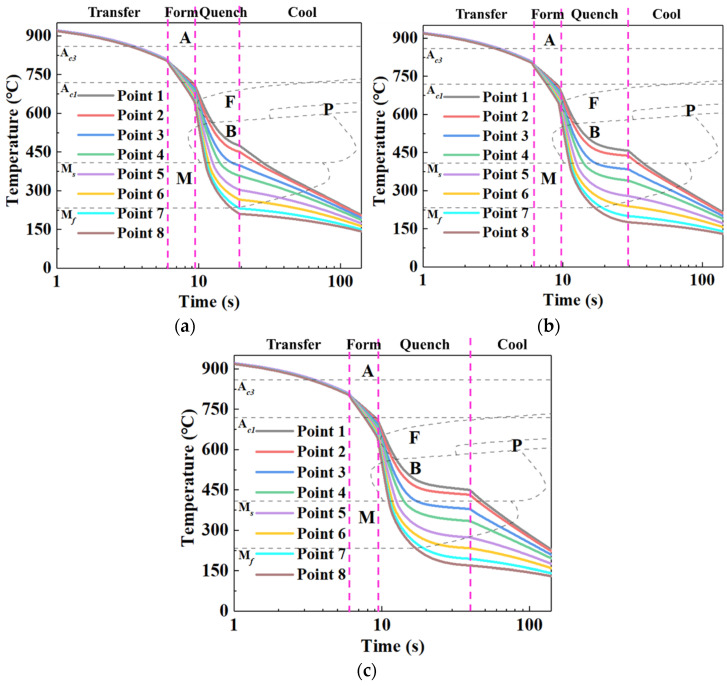
Temperature-history curves for sheet at eight temperature measuring points under different quenching times of (**a**) 10 s, (**b**) 20 s, and (**c**) 30 s.

Figure 9 exhibits the temperature history curves for sheet at eight temperature measuring points under different quenching times of 10 s, 20 s, and 30 s. It can be seen from Figure 9 that the curves can be divided into four stages, namely transferring, forming, quenching, and cooling. In Figure 9, A represents the austenite zone, F represents the ferrite transformation zone, B represents the bainite transformation zone, M represents the martensite transformation zone, and P represents the pearlite transformation zone. During the transferring stage, the temperature of the sheet (P1 to P8) uniformly dropped. During the forming stage, the temperature of the sheet began to differentiate due to the temperature gradient of the die, and an obvious feature of the temperature gradient was gradually formed. During the quenching stage, the temperature differentiation and temperature gradient of the sheet were more notable. It is worth emphasizing that, since the heat exchange between the sheet and die gradually slowed with the temperature of sheet decreasing, the temperature gradient of the sheet remained relatively constant with the further increasing of the quenching time. During the cooling stage, the temperature of the sheet dropped with the increasing time. Comparing the temperature-history curves at different quenching times, we found that the temperature of the sheet (P1 to P8) exhibited the same variation trend under different quenching times, and the cooling rate of the sheet (P1 to P8) was extremely slowed after quenching for 10 s, which is equivalent to the heat-preservation process. Nevertheless, it was worth noting that, in the quenching stage, for P1 to P8, the time of the phase transition increased with the increasing quenching time, which significantly affected the sufficiency of non-diffusive transformation.

**Figure 10:**
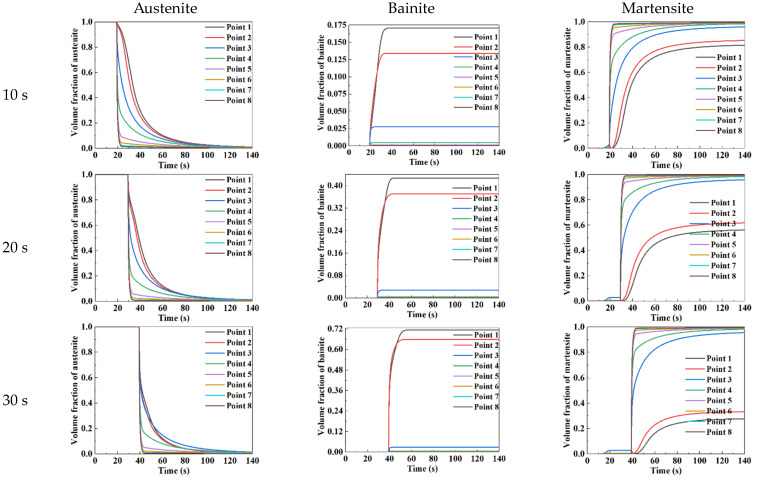
Variation of volume fraction of austenite, bainite, and martensite with time under different quenching time.

The curve of microstructural constituents, including austenite, martensite, and bainite, of the sheet (P1 to P8) over time is plotted in Figure 10. It was known from Figure 10 that the microstructure of the sheet (P1 to P8) was austenite before the quenching stage. The reason was that the temperature of the sheet dropped slowly under air-cooling, and there was no phase transformation. As can be seen from Figure 9 and Figure 10, during the quenching stage, the temperature curves of P1 and P2 were in the bainite transformation zone, resulting in the transformation from austenite to bainite occurring and the volume fraction of bainite increasing. The temperature curves of P3 to P8 were in the martensitic transformation zone due to the large cooling rate, leading to the transformation from austenite to martensitic occurring and the volume fraction of martensite increasing. Comparing the variation curves of the volume fraction of microstructures over time at different quenching times, it was obviously found that the volume fractions of austenite, bainite, and martensite exhibited the same variation trend under different quenching times. Furthermore, with the increase in quenching time, the longer time of the temperature curves of P1 and P2 passed through the bainite transformation zone, resulting in the transformation from austenite to bainite completely occurring, and the volume fraction of bainite was higher.

**Figure 11:**
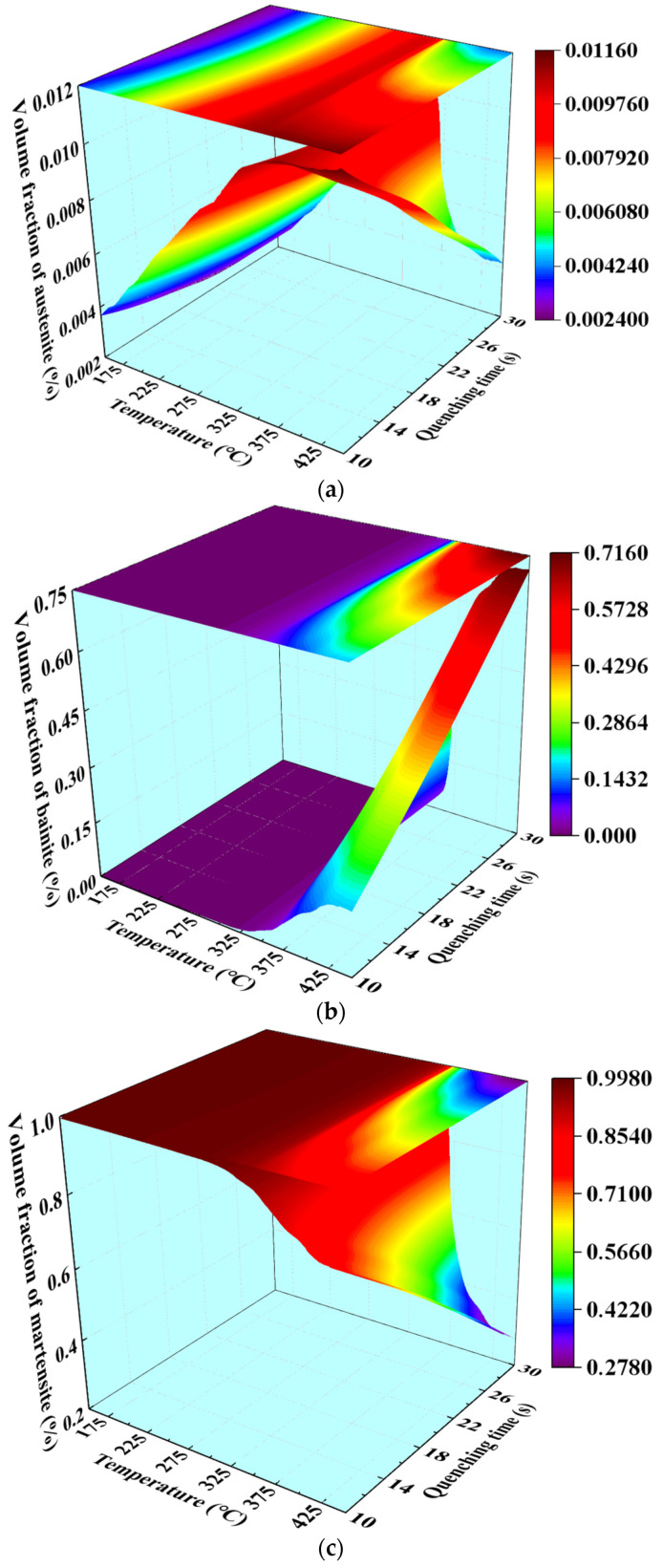
Three-dimensional mapping surfaces of (**a**) austenite, (**b**) bainite, and (**c**) martensite, with quenching time and the temperature of the die.

The distribution of the final volume fraction of austenite, bainite, and martensite along the length of sheet with quenching time and the temperature of die was characterized as a 3D mapping surface, as shown in Figure 11. It is evident that the volume fraction of microstructures was governed by the synergistic effect of quenching time and temperature. It can be summarized from Figure 11 that, when the temperature of the die was less than 325 °C, the volume fraction of martensite and bainite remained almost constant, and the volume fraction of austenite increased with increasing the temperature of the die. When the temperature of the die was higher than 325 °C, the volume fraction of martensite and austenite decreased with the increases of the quenching time and temperature of the die, while the volume fraction of bainite increased. Under the higher temperature of the die and lower quenching time, the volume fraction of austenite reached the maximum, which was 1.16%. The volume fraction of bainite reached the maximum of 71.6% under the higher quenching time and the higher temperature of the die. In addition, the summit of the volume fractions of martensite was 99.8%, corresponding to the temperature of the die below 350 °C.

### 3.2. Microstructure Characterization and Mechanical Property Test

**Figure 12:**
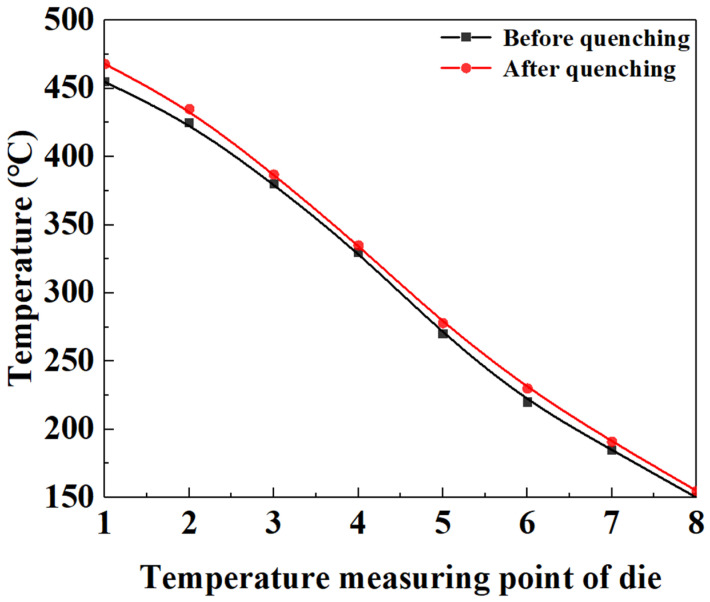
Temperature distribution of eight temperature measuring points of die before and after quenching.

In order to characterize and test the microstructure and mechanical properties of sheet under different quenching conditions and further explain the formation mechanism of gradient microstructure, the hot-stamping experiment of BR1500HS was conducted. Figure 12 shows the temperature distribution of eight temperature measuring points of the die before and after quenching. It can be observed from Figure 12 that the temperature distribution of the die presents a gradient characteristic, which meets the temperature requirements of the designed die.

**Figure 13:**
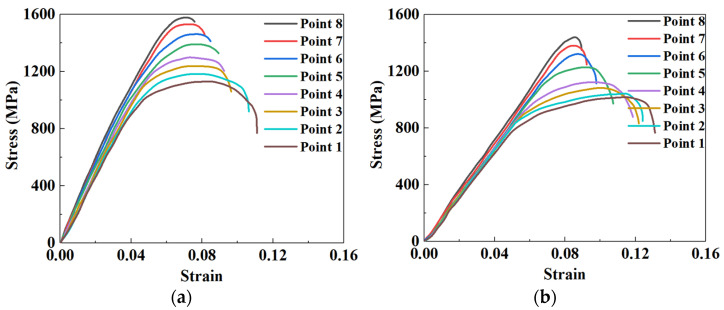

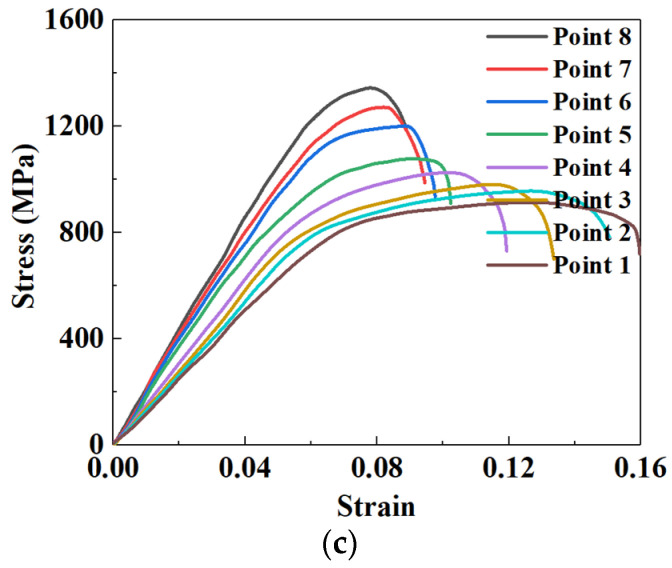
Stress–strain curves of different tensile specimens under the quenching time of (**a**) 10 s, (**b**) 20 s, and (**c**) 30 s.

The stress–strain curves from the tensile test at the corresponding temperature measuring point of sheet were shown in Figure 13. It can be seen from Figure 13 that the stress–strain curves for P8 to P1 present gradient distribution characteristics under the same quenching times. For all points, the peak stress successively decreases from P8 to P1, and the stress increases rapidly with the increase of strain first, and then decreases slowly. As for a fixed point, the peak stress decreases with the increasing quenching time.

In order to test the tensile strength, hardness, and elongation at break of different temperature measuring points of sheet, the tensile tests and microhardness tests for specimens were carried out using an electromechanical universal testing machine and HVS-1000 Digital microhardness tester, respectively. The results of tests were listed in Table 4. From Table 4, for P1 to P8, we can see that the tensile strength and hardness increased in turn, while the elongation at break decreased in turn at the same quenching time. Moreover, for the same point, the tensile strength and hardness decreased with increasing quenching time, and the elongation at break increased.

**Figure 14:**
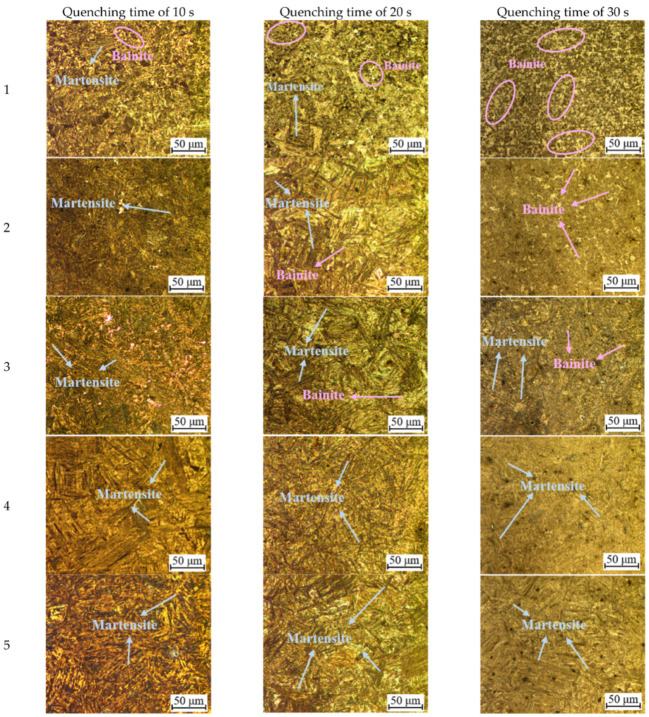

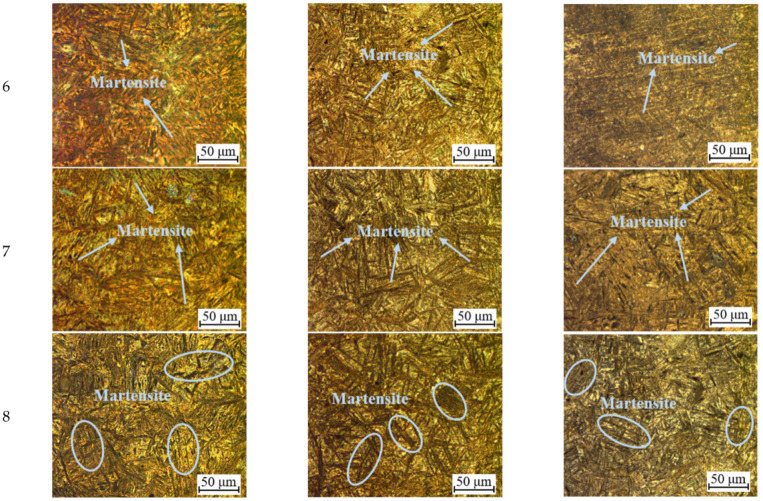
Metallography microstructures of different temperature measuring points.

Figure 14 presents the microstructures of sheet (P1 to P8) under the quenching times of 10 s, 20 s, and 30 s, respectively. As shown in Figure 14, the shape of martensite is lath, and the macroscopical dimension of martensite increases with increasing the temperature of die. The shape of bainite presents a gradual characteristic that bainite gradually changes from granular to double lenticular at a quicker cooling rate. It can be observed from Figure 14 that the martensite content increases with the increasing temperature of the die, and the bainite content decreases. On the other hand, as the quenching time increases from 10 s to 30 s, the martensite content of the sheet decreases, and the bainite content increases successively. At the same quenching time, the macroscopical dimension of martensite and bainite gradually becomes fine and dispersed. It can be summarized from Figure 14 that the required microstructures can be obtained by controlling the quenching time and the temperature of the die.

### 3.3. Construction of the Relationship of Quenching Parameter and Mechanical Property

**Figure 15:**
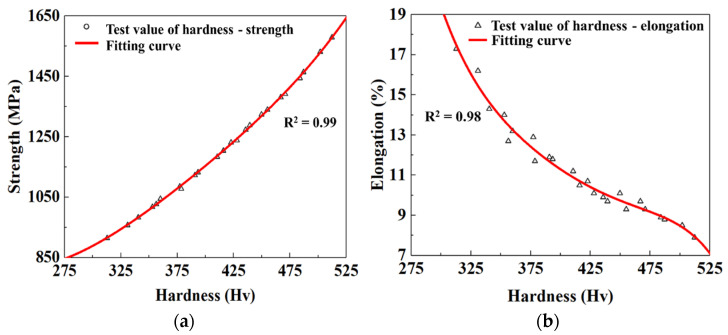
Fitted relationships of (**a**) hardness and tensile strength, and (**b**) hardness and elongation at break.

The mechanical properties of materials, including the tensile strength, hardness, and elongation at break, were characterized. Generally, strength, elongation, and hardness are related, and the hardness test is more convenient. In order to accurately estimate the strength and elongation of components through hardness, the test results of tensile strength, hardness, and elongation at break were counted, and then the fitted curves of hardness and tensile strength, and hardness and elongation at break were plotted by polynomial fitting, respectively, as shown in Figure 15. It can be noted from Figure 15 that tensile strength and hardness show a nonlinear positive relationship, while elongation at break and hardness shows a nonlinear negative relationship. The correlation coefficients of the fitted curves are greater than 0.98, thus proving that the fitted curves have higher precision and coherence.

**Figure 16:**
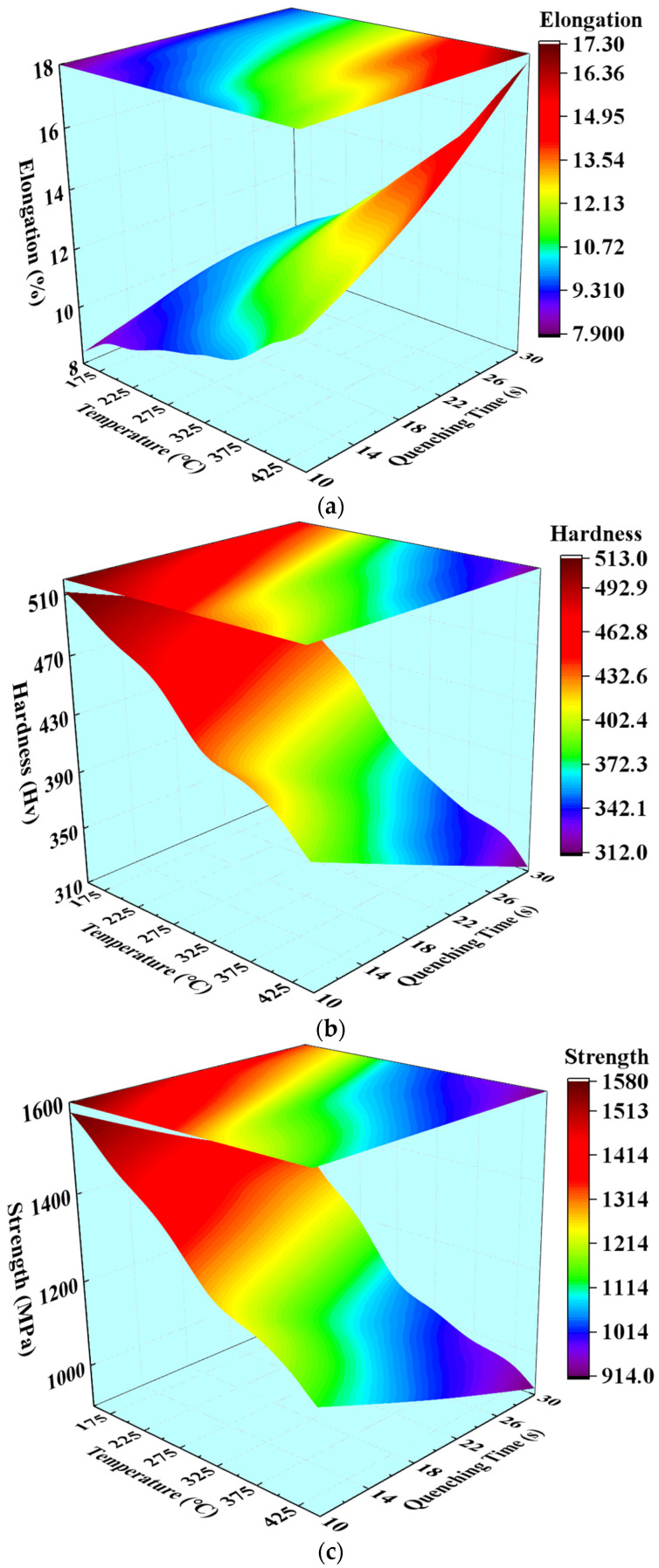
Mapping surfaces of (**a**) elongation at break, (**b**) hardness, and (**c**) tensile strength, which varied with the quenching time and the temperature of the die.

When the relationship between the quenching parameters and mechanical properties are known, a quenching scheme can be designed according to the requirements of the mechanical properties. According to the test results of mechanical properties in Table 5, three mapping surfaces between quenching parameters and mechanical properties can be fitted by the interpolation method, which significantly assists to control the mechanical properties during a hot-stamping quenching process. Figure 16 shows the mapping surfaces of elongation at break, hardness, and tensile strength varying with the quenching time and the temperature of die during the quenching process of BR1500HS. It can be observed from Figure 16 that the hardness of the sheet presents a gradient increasing trend with the decrease of quenching time and the temperature of the die. The variation of the tensile strength is consistent with that of hardness, while the variation of elongation at break is opposite to that of hardness. The elongation at break reaches the maximum of 17.3% under the higher quenching time and the higher temperature of the die. The summits of tensile strength and hardness are 513 HV and 1580 MPa, respectively, which corresponds to the lower quenching time and the temperature of the die. Combined with Figure 16 and Figure 11, it can be observed that the sheet located in the zone of the lower die temperature has higher strength and hardness. The reason is that the cooling rate of sheet is large, and the martensite transformation is dominant at this time. Then the full martensitic structure with high strength was obtained after cooling. When the die temperature is high, the cooling rate of sheet is also slow, and the sheet is in the bainite transformation zone. With the increase of quenching time, the bainite transformation time is also increasing, and the bainite structure with high toughness is finally obtained. These mapping surfaces not only revealed the corresponding mechanism between microstructures, mechanical properties, and quenching parameters of BR1500HS during the quenching process, but also can be employed to guide the hot-stamping process design, aiming to obtain the desired mechanical properties of components.

## 4. Design and Simulation of B-Pillar for Hot Stamping

### 4.1. Parameter Design and FE Model Construction of B-Pillar

In this work, the hot-stamping process for the automobile B-pillar with tailored mechanical properties was investigated, and its quenching process was designed according to the constructed mapping relationship. Generally, the bottom and top of the B-pillar were usually designed as an energy absorption zone, which requires mechanical properties with appropriate strength and elongation to pursue stronger crash energy-absorption ability. The middle zone was usually designed as an invasion-resistant zone, which requires a high strength to pursue an impact-resistant ability. Meanwhile, it is better to have a performance transition zone between the high- and the low-strength zones to prevent the failure caused by the sudden change of mechanical property.

**Figure 17:**
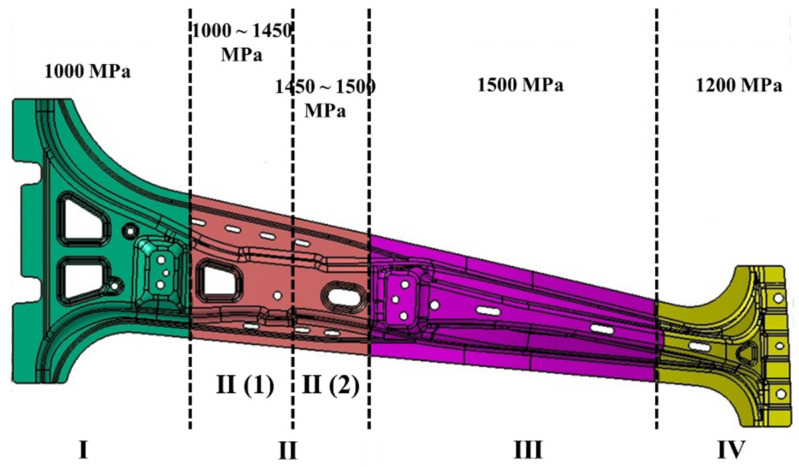
Segments of B-pillar with desired mechanical properties.

The gradient mechanical property zone of the B-pillar was shown in Figure 17. In Figure 17, the B-pillar was divided into four zones: (I) the energy absorption zone at the bottom, (II) the gradient performance zone between the energy absorption zone and the high-strength zone, (III) the high-strength zone at the middle, and (IV) and the energy absorption zone at the top. The tensile strength of III was 1500 MPa, and the tensile strength of zones I and IV was 1000 MPa and 1200 MPa, respectively. The tensile strength of II was 1000~1500 MPa, which can be subdivided into II (1) the main gradient zone dominated by bainite and martensite mixed microstructures and II (2) the weak gradient zone dominated by martensite (martensite content >95%, tensile strength >1450 MPa). Combined with the zone performance analysis and the mapping surface between the strength and quenching parameters, the quenching temperatures of different zones of the B-pillar were determined, as listed in Table 5.

**Figure 18:**
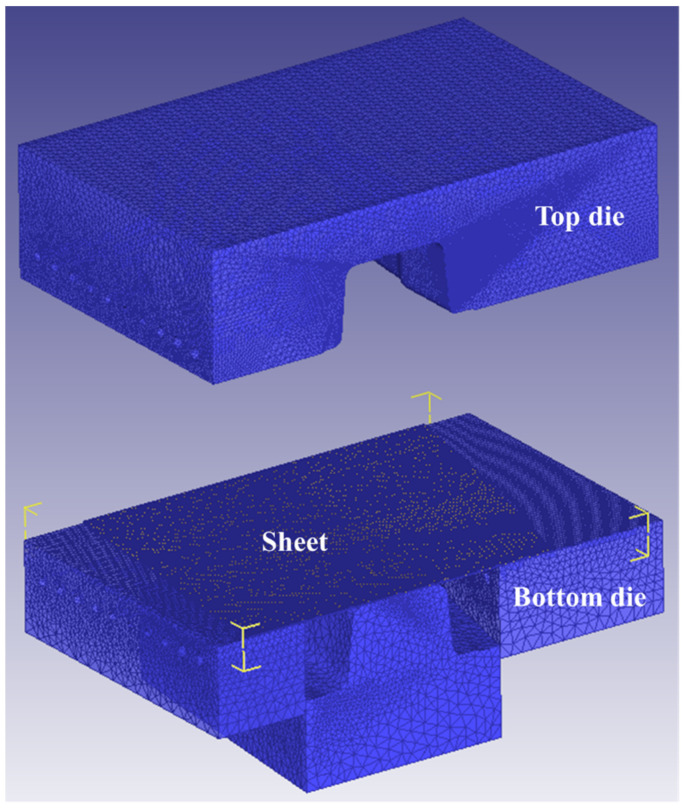
Finite-element model for gradient region of B-pillar.

The FE method was used to uncover the quenching process in DEFORM-3D software. Since the gradient performance zone has the characteristic of variable strength, the evolution mechanism of mechanical performance can be efficiently and accurately revealed by studying this zone. Therefore, the gradient performance zone of the B-pillar was extracted, and then the hot-stamping FE simulation of the automobile B-pillar was developed, as shown in Figure 18. The heating temperature of the gradient performance zone was 950 °C, the friction coefficient was 0.3, the transfer time was 9 s, the holding pressure was 3 MPa, and the quenching time was 20 s. The speed of the die was 60 mm/s in the fast-closing stage, and 30 mm/s in the slow-forming stage after blanking. H13 die steel was selected as die material from the material parameters in the Deform material library.

### 4.2. Analysis of Simulation Results

**Figure 19:**
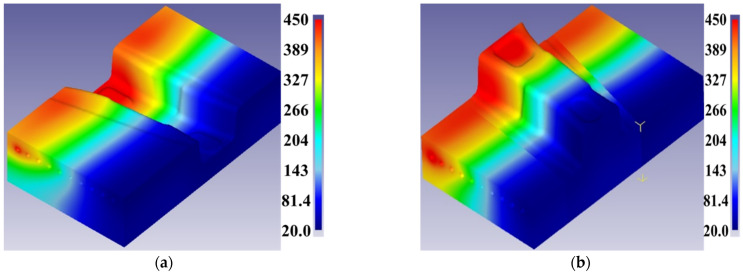
Temperature distribution of (**a**) top die and (**b**) bottom die.

In order to reveal the formation mechanism of the gradient performance zone, the temperature distribution of hot-stamping top and bottom dies must be analyzed first. The temperature distributions of the top and bottom dies are shown in Figure 19. It can be seen from Figure 19 that the temperature of the top and bottom dies presented the obvious characteristic of gradient distribution along the length of the die, gradually decreasing from 450 °C to 50 °C. The temperature of the weak gradient zone and main gradient zone was 150 °C to 50 °C and 450 °C to 150 °C, respectively.

**Figure 20:**
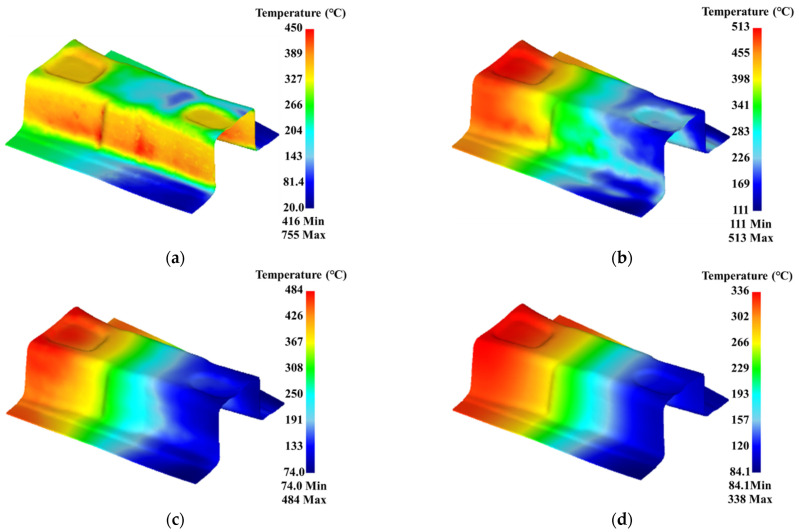
Temperature distribution with time for gradient region of B-pillar: (**a**) time of 14.6 s, (**b**) time of 24.6 s, (**c**) time of 34.6 s, and (**d**) time of 64.6 s.

The hot stamping of B-pillar can be divided into four stages: the transfer stage was 0–9 s, the forming stage was 9.3–14.6 s, the quenching stage was 14.6–34.6 s, and the cooling stage was 34.6–115 s. Four moments were selected for analysis, namely the end of the forming (time was 14.6 s), the quenching time of 10 s (time was 24.6 s), the quenching time of 20 s (time was 34.6 s), and the cooling time of 30 s (time was 64.6 s). The temperature distributions of the gradient performance zone at different times were shown in Figure 20. It is found from Figure 20 that, during the forming stage, the temperature of different positions of the B-pillar gradually appears to differentiate because of the different contact positions and contact times between the B-pillar and die. When the forming was finished, the temperature of the side wall was higher, followed by the concave zone at the top, and the temperature of the flange zone, with the longest contact time with the die, was the lowest. Because the sheet deformation of the side wall was large, and a lot of heat was generated through friction, the temperature was higher than it was in other zones. When the quenching time was 10 s, the gradient temperature of the B-pillar was gradually formed under the action of the gradient temperature of the die. When the quenching time was 20 s, the gradient temperature of the B-pillar was observably formed because of the long-time contact heat transfer between the die and the sheet for a long time. During the cooling stage, the temperature of the B-pillar dropped with the increasing time. The sheet evenly cooled at room temperature, and the temperature gradient of each zone was gradually decreasing.

**Figure 21:**
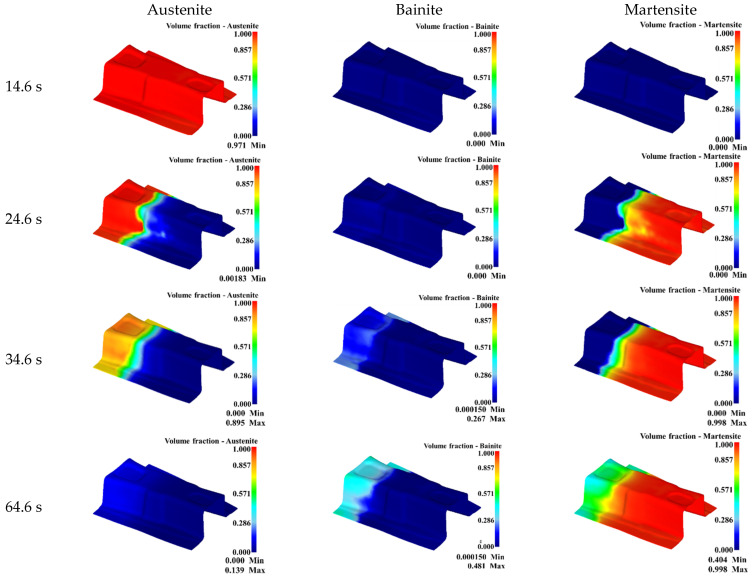
Volume fraction of austenite, bainite, and martensite for gradient zone of B-pillar.

The volume fractions of austenite, bainite, and martensite at different times in the gradient performance zone were displayed in Figure 21. It can be seen from Figure 21 that the B-pillard only underwent a heating and heat-preservation process during the forming stage, during which there was no phase transition. When the quenching time was 10 s, the temperature of the weak gradient zone dropped rapidly into the martensite transformation zone under the action of high die temperature, and martensite transformation occurred. The temperature of some main gradient zone dropped slowly into the bainite transformation zone, and bainite transformation occurred. Meanwhile, the obvious microstructure transition zone could be seen between the main gradient zone and the weak gradient zone. When the quenching time was 20 s, the martensite transformation was basically completed in the weak gradient zone, and the bainite transformation in the main gradient zone was gradually filled with the increase of the quenching time. The maximum of the bainite content in the main transition zone reached 26.7%. During the cooling stage, the main transition zone within the bainite transformation zone continued to carry out the transformation from austenite to bainite with the increasing cooling time. When the time was 64.6 s, the temperature of the main transition zone decreased to the temperature of the martensite transition zone, and bainite transformation ended, with the maximum bainite content of 48.1%. In the main gradient zone, the austenite without martensitic transformation was in the martensitic transformation zone, and then martensitic transformation occurred. The microstructure in the weak gradient zone was almost entirely martensite with high strength, while the main gradient zone was a mixture of martensite and bainite, which has high energy-absorption characteristics.

**Figure 22:**
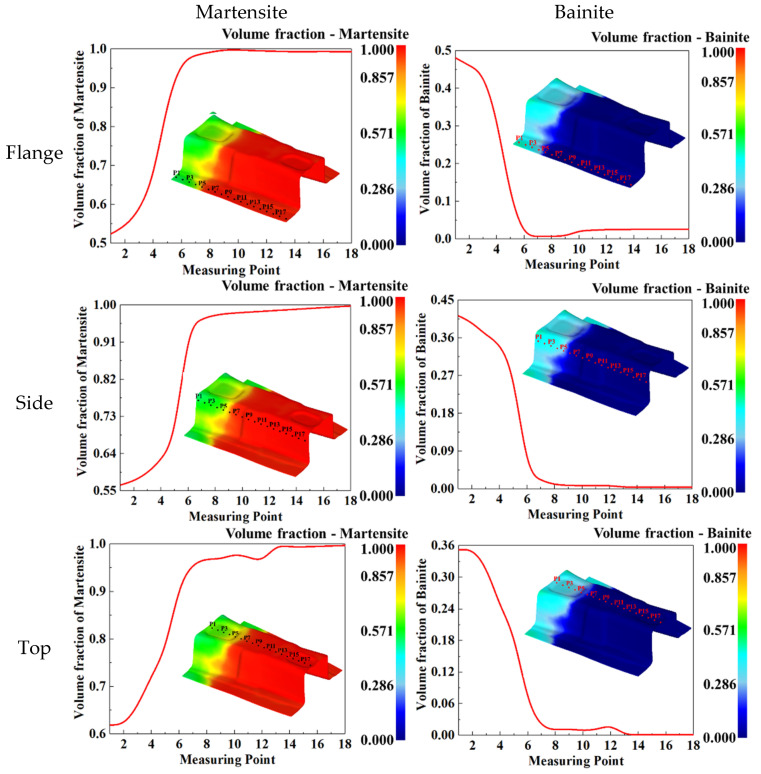
Final microstructures volume fraction of three typical positions: flange, side wall, and top.

In addition, during the hot-stamping process, the contact time between the B-pillar and die at different positions was different, resulting in different phase transformation processes and final microstructures. Therefore, the final microstructure distribution of three typical parts, namely the flange edge, side wall, and top of the B-pillar, were studied. In order to study the volume fractions of the distribution of bainite and martensite in detail, 18 tracking points were uniformly selected from the flange edge, side wall, and top of the B-pillar, and then the volume fraction curve of bainite and martensite over tracking point was drawn, as shown in Figure 22. As can be seen from Figure 22, in the side wall of B-pillar, the volume fraction of martensite was small at P1 to P4, and it increased rapidly from P4 to P7, and then it reached stability. On the contrary, the variation of the volume fraction of bainite was large at P1 to P4; it rapidly decreased from P4 to P7, and then it reached stability. The variation of volume fraction of martensite and bainite at the flange edge and top of B-pillar was the same as that of side wall. In addition, for the same tracking point, the volume fraction of bainite at the flange edge was the largest, and at the top, it was the smallest; the volume fraction of martensite at the flange edge was smallest, and at the top was the largest. The reason is that the contact time between the die and the sheet was longer, and the time of bainite transformation was more sufficient. It is worth noting that the curve of the volume fraction over the tracking point at the top is not smooth. This is because the top of B-pillar is not flat and has the characteristics of a basin, which affects the temperature change during the quenching process, and then affects the change of microstructure. The simulation results of B-pillar were highly consistent with the abovementioned simulation results of sheet, which can predict the mechanical properties of components. It was confirmed that the mapping relationship of the quenching parameters and the phase and mechanical properties was beneficial and practical to design quenching parameters aiming at different serving conditions in practice.

## 5. Conclusions

The effect of quenching time and the temperature of die on the phase transformation characteristics of BR1500HS was revealed with the help of finite element simulation, and the mapping relationships of quenching time, temperature and each phase were successfully constructed. The relationship curves between hardness and tensile strength, and hardness and elongation at break were fitted, respectively, and then the mapping surfaces of quenching time, the temperature die, and mechanical properties were constructed. It is applied for quenching parameters’ design, with the aim of making a local adjustment of the phase and mechanical properties. The main conclusions of this work are summarized as follows:

(1) The bainite transformation gradually predominates with the longer quenching time in the die’s high-temperature zone, and the martensitic transformation predominates in the die’s low-temperature zone. Moreover, the relationships of the temperature die, quenching time, and each phase were characterized as a 3D mapping surface.
(2) The relationship curves of hardness and tensile strength, and hardness and elongation at break were fitted, respectively; it was shown that the tensile strength increases with the increasing hardness, and the elongation at break decreases.
(3) Three 3D mapping surfaces are constructed to clarify the corresponding relationships of hardness, tensile strength, and elongation at break, varying with the temperature die and quenching time. Tensile strength and hardness increase with decreasing quenching time and the temperature die, and the elongation at break decreases.
(4) The quenching parameters of the B-pillar in different serving conditions were designed, and an FE simulation was carried out to verify. The verification results indicated that the required phase and mechanical properties of BR1500HS were achieved by using the designed quenching parameters. This design principle of the processing parameters also can contribute to the local adjustment of phase and mechanical properties for other steels.

## Figures and Tables

**Table 1:** Composition of BR1500HS sheet metal used in this work (wt.%).

C	Si	Mn	Cr	Ni	P	Mo	S	B	Al	Ti	Cu	V	Fe
0.23	0.25	1.35	0.19	0.028	0.015	0.04	0.006	0.003	0.04	0.03	0.016	0.004	balance

**Table 2:** Experiment scheme of BR1500HS in the hot stamping.

Temperature Gradient of Die	Initial Forming Temperature	Quenching Time
450–150 °C	800 °C	10 s
450–150 °C	800 °C	20 s
450–150 °C	800 °C	30 s

**Table 3:** Thermal physical property of BR1500HS.

Temperature (°C)	20	100	300	400	600	800	1000
Young’s modulus (GPa)	212	207	193	166	150	134	118
Poisson ratio	0.284	0.286	0.293	0.298	0.31	0.325	0.343
Thermal conductivity, (W/(m·°C))	34.1	36.3	36.7	32.8	35.6	38.2	39.6
Specific heat (J/(Kg·°C))	629	630	560	580	700	755	810

**Table 4:** Hardness, tensile strength, and elongation at break of sheet at different temperature measuring points.

Time	Property	1	2	3	4	5	6	7	8
10	Tensile strength (MPa)	1132.7	1183.5	1237.8	1288.2	1391.7	1464.2	1531.2	1579.1
	Hardness (Hv)	393.3 ± 2.5	410.4 ± 1.4	427.9 ± 2.7	439.2 ± 3.2	470.7 ± 3.3	486.9 ± 4.1	501.7 ± 1.2	512.1 ± 3.9
	Elongation at break (%)	11.8	11.2	10.1	9.7	9.3	8.8	8.5	7.9
20	Tensile strength (MPa)	1017.9	1044.4	1085.3	1123.0	1230.9	1323.7	1380.8	1443.9
	Hardness (Hv)	352.8 ± 2.1	359.9 ± 2.6	377.1 ± 3.1	390.6 ± 1.7	422.6 ± 1.9	449.6 ± 3.4	466.7 ± 2.1	483.8 ± 2.1
	Elongation at break (%)	14.0	13.2	12.9	11.9	10.7	10.1	9.7	8.9
30	Tensile strength (MPa)	914.8	957.6	983.0	1026.8	1077.7	1204.0	1273.3	1339.6
	Hardness (Hv)	312.7 ± 3.2	330.8 ± 2.2	340.2 ± 2.5	356.2 ± 3.7	378.5 ± 2.1	415.8 ± 2.8	435.6 ± 1.7	454.9 ± 1.5
	Elongation at break (%)	17.3	16.2	14.3	12.7	11.7	10.5	9.9	9.3

**Table 5:** Simulation scheme of B-pillar.

Segment	Tensile Strength (MPa)	Temperature of Die (°C)
I	1000	450
II (1)	1000~1450	150~450
II (2)	1450~1500	50~150
III	1500	50
IV	1200	280

## Data Availability

Not applicable.
